# A Study of the Long-Term Electrochemical Stability of Thin-Film Titanium–Platinum Microelectrodes and Their Comparison to Classic, Wire-Based Platinum Microelectrodes in Selected Inorganic Electrolytes

**DOI:** 10.3390/ma17061352

**Published:** 2024-03-15

**Authors:** Zbigniew Szklarz, Karolina Kołczyk-Siedlecka, Elizaveta Vereshchagina, Aina Herbjørnrød, Paul Wittendorp, Shruti Jain, Pawel Jerzy Wójcik

**Affiliations:** 1 Department of Microfluidic Electrochemistry, Redoxme AB, 30-059 Kraków, Poland; 2 Faculty of Foundry Engineering, Department of Chemistry and Corrosion of Metals, AGH University Krakow, al. A. Mickiewicza 30, 30-059 Kraków, Poland; 3 Faculty of Non-Ferrous Metals, Department of Physical Chemistry and Metallurgy of Non-Ferrous Metals, AGH University Krakow, al. A. Mickiewicza 30, 30-059 Kraków, Poland; 4 Department of Smart Sensors and Microsystems, SINTEF Digital, Gaustadalléen 23C, 0373 Oslo, Norway; elizaveta.vereshchagina@sintef.no (E.V.); aina.k.herbjornrod@sintef.no (A.H.); paul.wittendorp@sintef.no (P.W.); shruti.jain@sintef.no (S.J.); 5 Research & Development Department, Redoxme AB, Södra Grytsgatan 1A, 7226-3005, 602 33 Norrköping, Sweden; pawel.wojcik@redox.me

**Keywords:** microelectrodes, cyclic voltammetry, thin-film electrochemical electrodes

## Abstract

This paper discusses the electrochemical properties of thin-film, planar, titanium–platinum (Ti-Pt) microelectrodes fabricated using glass or silicon substrates and compares their performance to the classic platinum (Pt) microelectrodes embedded in glass. To analyze the possible differences coming both from the size of the tested electrodes as well as from the substrate, short- and long-term electrochemical tests were performed on selected water electrolytes (KCl, HCl, KOH). To study the electrochemical response of the electrodes, the cyclic voltammetry (CV) measurements were carried out at different scanning rates (from 5 to 200 mV/s). Long-term tests were also conducted, including one thousand cycles with a 100 mV/s scan rate to investigate the stability of the tested electrodes. Before and after electrochemical measurements, the film morphology was analyzed using a scanning electron microscope (SEM). The good quality of the thin-film Pt electrodes and the high repeatability in electrochemical response have been shown. There are minor differences in standard deviation values taken from electrochemical measurements, comparing thin-film and wire-based electrodes. Damages or any changes on the electrodes’ surfaces were revealed by SEM observations after long-term electrochemical tests.

## 1. Introduction

Electrochemical methods have been known for decades and are still often used [1]. Miniaturization in electrochemistry helps to circumvent mass transfer limitations when the diffusion rate is lower than the rate of electrochemical reactions, with a high impact in the case of high-speed reactions. Miniaturization of electrochemical cells improves mass transport [2]. Integrated thin-film microelectrodes can be used in the analysis of materials, reactions or solutions, detection [3,4,5,6], sensors [7,8], biosensors [1], bioelectrochemistry [9], and microfluidics [1]. Reducing the dimensions, specifically the thickness of the electrodes, to tens of nanometers may result in differences in the electrodes’ performance (e.g., sensitivity, lower capacitance, faster response, etc.) as nanoscale effects prevail. Nanoelectrodes can be used for electrochemical analysis of single cells, single enzymes, and single nanoparticles [10,11,12].

Platinum electrodes are still widely used in electrochemistry mainly because of their catalytic properties. However, Pt is an expensive metal; thus, production of thin but good-quality electrodes may be an efficient method to obtain cheaper analytical tools as well as components for other applications (energy conversion, for example) [13,14,15,16,17]

Microelectrodes often exhibit comparable behavior to the electrodes on the macroscale. Jacobse et al. compared Pt micro- and macroelectrodes in the reactions of [Ru(NH_3_)_6_]^3+^ reduction and FcCH_2_OH oxidation [18]. The activity of these reactions carried out on microelectrodes was observed to agree with the annealed macroelectrodes. The slightly higher slope of the Tafel curve was explained by faster diffusion to and from the microelectrodes.

The set of microelectrodes intended for multiple measurements is called microelectrode arrays (MEAs). Depending on the type of substrate, MEAs can be made rigid and flexible [19]. In addition, depending on the geometry of substrates and the arrangement of microelectrodes, MEAs are divided into two-dimensional (2D) and three-dimensional (3D) [20,21]. Two-dimensional rigid MEAs were the first to be developed and are still extensively studied [22]. Thin-film electrodes are commonly deposited using magnetron sputtering and evaporation methods on substrates such as glass [23], silicon [24], and ITO-coated glass [25]. Among reported flexible electrodes are Au on parylene-C [26], polyimide [24], or PDMS [27,28]. Hence, there are many possible applications for thin film 2D electrodes, such as measurement systems or sensors of any geometry.

Process analysis in the flow has become more common with the development of technologies for integrating microelectrodes in miniaturized flow cells [29,30,31,32]. Integrated microfluidic, spectroelectrochemical cells open for the development of materials for analysis in continuous flow systems, which can be further applied, for example, in sensors [33,34,35]. The critical design parameters are relative dimensions of integrated microelectrodes and microchannels, allowing for controlled, bubble-free flow of electrolytes and other reagents. Additionally, deposition techniques for thin metal films should yield electrodes with high electrochemical stability suitable for long-term use.

The thin-film microelectrodes have many advantages, e.g., the possibility of using electrodes in different and complex geometries that allow for additional surface modifications. The electrodes fabricated using Si technology can be integrated with other miniaturized sensors and actuators and microfluidic systems for sample preparation and handling, extending the range of possible applications.

In this work, the electrochemical properties of thin-film Ti-Pt microelectrodes deposited on boro-float glass and oxidized silicon (Si) were analyzed and compared with the properties of Pt wire-based microelectrodes embedded in the glass tube. KCl, HCl and KOH—the most common environments in inorganic chemistry—were selected for the experimental study. The aim of this work was to determine the electrochemical stability and electrical signal repeatability of the thin-layer microelectrodes. This is the first stage of our research, and those glass and silicone chips will be used for microfluidic systems. Short- and long-term tests in stationary conditions were made to analyze possible differences in electrochemical response coming from the substrate as well as differences comparing to wire-based Pt electrodes.

The electrodes were analyzed using a cyclic voltammetry method (CV). The surface morphology was assessed before and after the electrochemical tests using a scanning electron microscope (SEM). The characteristic peaks in the electrolytes, their linearity depending on the scan rate, and the repeatability of the obtained results were analyzed. The results suggest a high potential for using the analyzed microelectrodes in the analysis of various electrochemical reactions, including redox reactions, gas generation, metal electrodeposition and dissolution, or oxidation and reduction of organic compounds. In addition, long-term measurements were carried out by taking 1000 CV measurements at a given scan rate to determine the stability of the electrodes. 

## 2. Experimental

### 2.1. Materials

All reagents were characterized by analytical purity (WARCHEM sp. z o.o., Zakręt, Poland). Demineralized water (WARCHEM sp. z o.o., Zakręt, Poland).

### 2.2. Wire-Based Pt Microelectrodes

**Figure 1:**
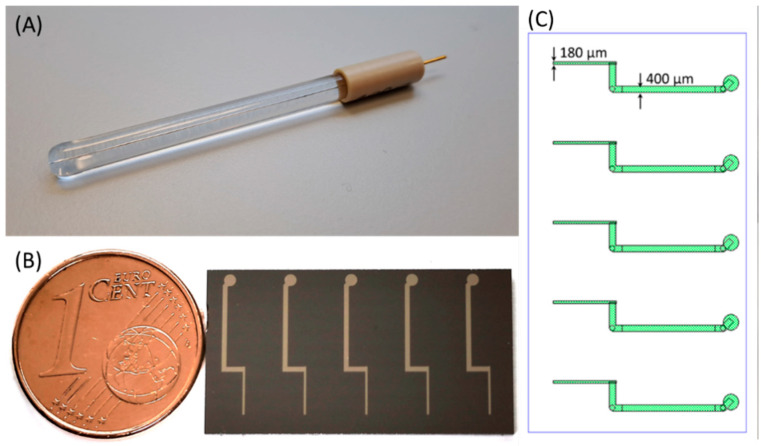
The photographs of: (**A**) wire-based Pt microelectrode (200 µm), and (**B**) example of an Si die with patterned thin-film Ti-Pt microelectrodes. (**C**) Design of electrodes.

Wire-based Pt microelectrodes in Figure 1A (Redox.me, Kraków, Poland) were used to obtain the background measurements for comparison with the microfabricated Ti-Pt electrodes. 

Three Pt microelectrodes were manufactured in the form of wires with different diameters i.e., 50, 100 and 200 µm, and embedded in the glass body exposing the wire cutting surface only (Figure 1A). After each series of measurements, the electrodes were polished with Al_2_O_3_ paste with a diameter of 0.05 µm and washed by demineralized water. To finish, the electrodes were placed for 10 min in demineralized water in an ultrasonic cleaner. The surface quality and the cleanliness of the electrodes were checked using an optical microscope.

### 2.3. Thin-Film Pt Microelectrodes

Two types of electrode substrates were prepared—on glass and Si (Figure 1B). They were 150 mm in diameter, double-side polished, 525 µm in thickness borofloat glass wafers and 150 mm in diameter, single-side polished, n-type Si wafers, and 675 µm in thickness. Prior to thermal oxidation (ca. 500 Å thermal oxide), the Si wafers were double-RCA-cleaned, and spin-dried. Prior to the deposition of thin metal films, both Si and glass wafers were cleaned in SC-1 cleaned for 10 min, followed by 10 min rinse in DI water and spin drying. The oxidized Si wafers were RCA-cleaned and rinsed in DI water for 10 min. After this, 15 nm of Ti adhesion layer and 100 nm of Pt were deposited on both types of wafers using an AMAT Endura cluster tool, in a single wafer chamber with DC magnetron configuration. The wafers were not clamped during sputtering, and there was no active temperature control of the wafer. A photoresist mask was used for patterning of Pt. The design of the electrode is shown in Figure 1C, with narrowest (180 µm wide) and widest (400 µm wide) parts indicated. Before coating with photoresist, 3 min plasma clean at 100 W was run. Then, 2.6 µm thick positive photoresist (HIPR 6512, Fujifilm, Tokyo, Japan) was spin-coated for patterning of Ti-Pt and soft-baked at 100 °C for 60 s. Wafers were exposed using an MA150 mask aligner developed in AZ 726 MIF developer (Merck, Rahway, NJ, USA) for about 60 s, hard-baked for 90 s at 130 °C, and plasma-cleaned at 100 W for 3 min before reactive ion etching (RIE) of Ti-Pt. The etching of Ti-Pt was carried out in a parallel-plate etcher PlasmaPro 80 RIE (Oxford Instruments, Abingdon, UK). After this, the resist was removed in Tepla 300 plasma ashing system during 5 min at 1000 W and 30 min at 550 W plasma. Further, the wafers were inspected in an optical microscope and diced using dicing saw Disco DAD321. Resulting chips had dimensions of ca. 13.85 × 25.5 mm^2^.

### 2.4. Electrochemical Measurements

The electrochemical behavior of electrodes was analyzed in neutral salt (KCl), acid (HCl), and alkaline (KOH) electrolytes. All three electrolytes were water-based solutions in 0.1 M concentration. 

**Figure 2:**
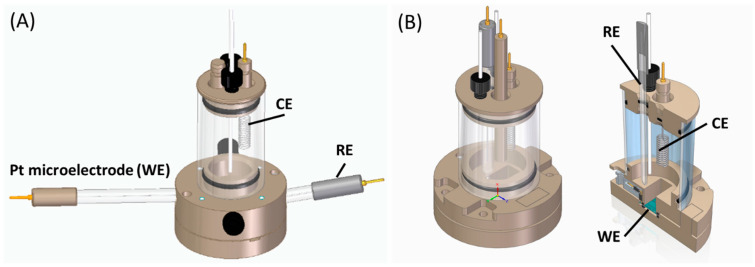
The electrochemical cells used for experiments on (**A**) wire-based Pt microelectrodes, (**B**) thin-film Ti-Pt microelectrodes.

Figure 2A,B present 3D schematic drawings of the three-electrode electrochemical cells (redox.me, Sweden) that were used for the tests of both wire-based and thin-film Ti-Pt microelectrodes. The body of the first cell (Figure 2A) is made of polyether ether ketone (PEEK), which has high chemical and mechanical resistances. The body of the second cell (2B) was SLA-printed using Rigid 10K Resin (Formlabs, Somerville, MA, USA). For all tests, Pt wire or thin-film Ti-Pt acted as a working electrode (WE), the platinum spiral wire was used as a counter electrode (CE), and the Ag/AgCl or Hg/HgO electrodes were employed as reference electrodes (RE) for chlorides or hydroxide electrolytes, respectively (all conventional electrodes supplied by redox.me, Norrköping, Sweden). In the case of thin-film Ti-Pt microelectrodes (Figure 2B), there were five inputs (WE) available; however, only one was tested at a time. The cells were tightly closed, limiting the access to the air. The exact amount of electrolyte was used in each measurement. The experiments were run at 20 °C to minimize the influence of the external environment on the obtained results. 

### 2.5. Measurement Methodology

To study the electrochemical stability and repeatability of microfabricated Pt electrodes, cyclic voltammetry (CV) was used. In this method, the WE sample is polarized alternately with a linearly increasing and decreasing potential within a selected range, allowing oxygen and hydrogen evolution to be observed. During the polarization, the current response and cyclo-voltammograms are acquired. The range of the potential window is set individually depending on the type of electrolyte. Electrochemical measurements were performed using the MULTI AUTOLAB M204 galvanostat-potentiostat and computers equipped with NOVA 2.1.5 software.

There were two stages of measurements: (i) the long-time measurements, where 1000 CVs with scan rate of 100 mV/s were applied to investigate the electrochemical stability and repeatability, and (ii) the scan rate effect, where the electrodes were polarized with different potential scan rates (there were 5, 10, 20, 50, 100 and 200 mV/s scan rates—thirty repetitions for each).

The microfabricated Ti-Pt electrodes were analyzed using the scanning electron microscopy (JEOL Microscope, Taiwan, China) method before and after the measurements to assess the surface condition and possible influence of the given electrolyte.

## 3. Results and Discussion

### 3.1. Electrochemical Response of the Wire-Based Pt Microelectrodes in Selected Electrolytes

**Figure 3:**
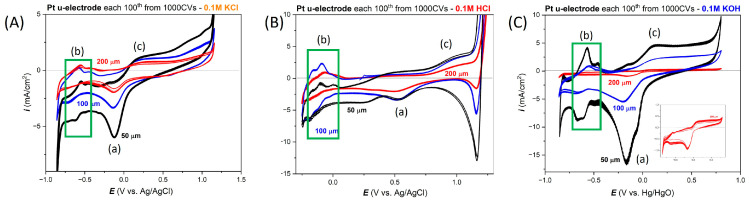
The electrochemical response from the wire-based Pt microelectrodes with the diameters 50, 100 and 200 um in (**A**) 0.1 M KCl, (**B**) 0.1 M HCl and (**C**) 0.1 M KOH electrolytes. For each diameter, the 1000 CVs have been registered in the selected electrolyte.

The wire-based Pt microelectrodes served as a reference to obtain the baseline electrochemical responses and to facilitate further comparison to the thin-film Pt electrodes. Figure 3 presents the CVs registered for the wire-based Pt microelectrodes with three diameters. These results have been obtained during long-term cycling, which included 1000 cycles. Only every 100th CV curve was plotted for the sake of clarity. The range of the potential window allowed us to observe both oxygen and hydrogen evolution, visible in the form of a sharp increase in the value of currents at the boundaries of the potential range. Moreover, the characteristic anodic and cathodic peaks are visible depending on the electrolyte type. For example, in KCl electrolyte (Figure 3A), at −0.12 V vs. Ag/AgCl, the irreversible peak of oxide desorption (a) is very clearly seen, the same as in KOH (at −0.16 V vs Hg/HgO, Figure 3C). In HCl acid, the current value for this peak is significantly lower (Figure 3C). There are also peaks from the hydrogen desorption (b) and irreversible peak, indicating the oxidation of the Pt surface (c). These results correspond well with those reported in the literature and show the typical response of Pt in the KCl, HCl and KOH [36,37]. 

The influence of the diameter of the Pt microelectrodes can be derived. When the diameter is smaller, the registered current values increase. This probably corresponds to the limited transport of substrates and products of electrochemical reactions in the vicinity of the electrode, which is a well-known phenomenon [38].

**Figure 4:**
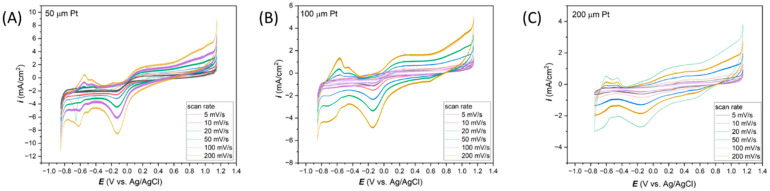
Effect of scan rates on the electrochemical response in 0.1 M KCl registered for the Pt microelectrodes with diameters (**A**) 50 µm, (**B**) 100 µm, and (**C**) 200 µm.

Figure 4 presents the dependence of the scan rate on the shape of CV curves depending on the diameter of the wire-based Pt microelectrodes. As before (Figure 3), the smallest electrode with a diameter of 50 µm is characterized by the highest current density values and, therefore, the sharpest peaks in CV curves.

The repeatability of the obtained results was determined based on the analysis of the current density in the characteristic peaks on the CV curves. The average current density and standard deviation for 30 cyclovoltammetry curves at a specific potential were determined.

Table 1 presents the calculations obtained for classic Pt microelectrodes of various diameters. The average current density was determined in the −0.18 V peak vs. Ag/AgCl (peak marked in Figure 3A–C). Differences in the average current densities depending on the scan rate are explained by the different transport rates of the components of electrochemical reactions. In addition, there are differences depending on the size of the electrode surface. Larger values of standard deviations are observed in the case of the lowest scan rate, reaching even several percent. Higher scan rates are associated with lower measurement errors.

The average standard deviation of the measurements made at all scan speed values for the specified electrodes was determined. The average value of the peak current density standard deviation is 1.43, 2.58 and 0.83% for Pt electrodes with a diameter of 50, 100 and 200 µm, respectively. 

To avoid this article being too long, the Supplementary Materials (SM) have been added just to show the electrochemical response of Pt wire-based in 0.1 M HCl and 0.1 M KOH electrolytes and compare them with those coming from Pt chips. 

As can be seen in SM, there is a similar effect of the electrode size (diameter) on the electrochemical response at low- and high-pH electrolytes (0.1 M HCl and 0.1 M KOH, respectively). For both HCl and KOH electrolytes, when the diameter is smaller, the registered current values increase. Moreover, cyclic voltammetry curves indicate high repeatability, regardless of the environment and scanning speed.

### 3.2. Electrochemical Response of Thin-Film Pt Microelectrodes in Neutral Salt (0.1 M KCl)

**Figure 5:**
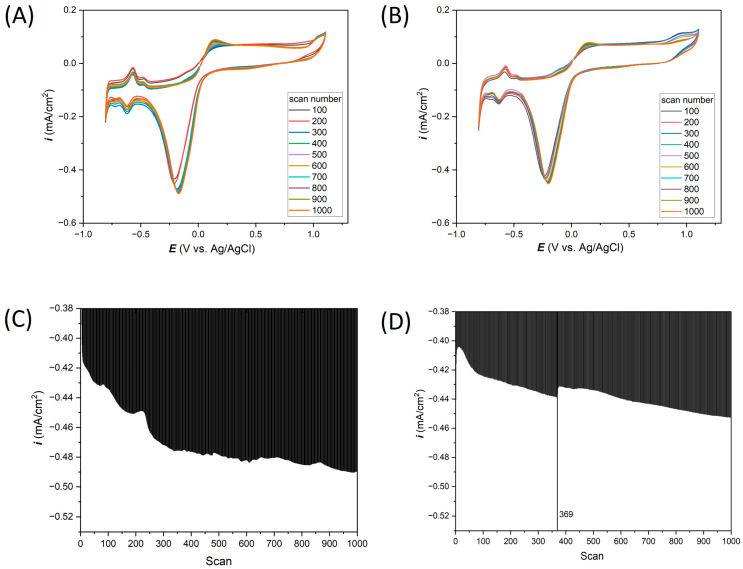
A total of 1000 cyclic voltammetry measurements in 0.1 M KCl prepared for (**A**) Ti-Pt on glass, (**B**) Ti-Pt on Si; and cathodic peaks at −0.18 V vs. Ag/AgCl analysis for (**C**) Ti-Pt on glass and (**D**) Ti-Pt on oxidized Si.

The thin-film Ti-Pt microelectrodes on glass and Si were tested in 0.1 M KCl solution. For measurements carried out in neutral salt environment, CV measurements were made in the potential range from −0.8 to 1.1 V vs. Ag/AgCl (Figure 5A,B, for Ti-Pt electrode on glass and Ti-Pt electrode on Si, respectively). The initial analysis of the 1000 CVs did not reveal any significant differences in electrochemical response, that could come from the different substrates. CV curves recorded on both types of substrates are characterized by the same shape and highly similar current densities. However, to be able to analyze the electrochemical response from Ti-Pt thin-film microelectrodes in more detail, the dependence of the current density value of peak at −0.18 V vs. scan number is presented in Figure 5C,D. A nearly continuous increase in the cathodic current density value was observed. Still, these values are small relative to the direct value of the current density recorded in the measurement. Some minor changes (fluctuations) in current density values can be observed, which indicates possible processes on the Pt surface, e.g., oxidation or the release of hydrogen or oxygen bubbles. Importantly, Figure 5A,B show the CV curves for the thin-film Pt on glass and Si substrates, respectively. The same shape of the curves can be observed as in the wire-based Pt microelectrodes (Figure 3A).

**Figure 6:**
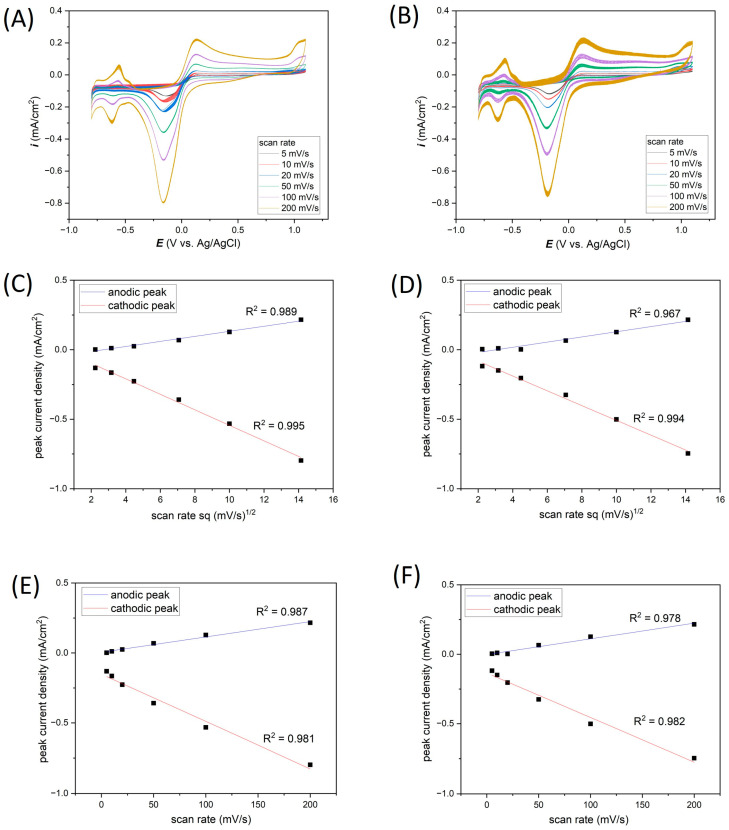
Effect of scan rates on the electrochemical response in 0.1 M KCl registered for (**A**) Ti-Pt on glass, (**B**) Ti-Pt on Si; and anodic/cathodic peaks linearity for (**C**,**E**) Ti-Pt on glass and (**D**,**F**) Ti-Pt on Si.

As expected, the CV curves’ dependance on the scanning speed have the same shape both in the case of measurements carried out on the Ti-Pt microelectrodes on glass and on Si (Figure 6A,B). 

In Figure 6C–F, the relationship between most characteristic cathodic current peaks at the potential of about −0.18 V and anodic current peaks at the potential of about 0.13 V vs. Ag/AgCl relative to the scanning speed for Pt on glass and Pt on Si have been plotted. From these graphs, the oxidation–reduction mechanism can be determined. The current peaks were read from 20th CV for all cases.

In Figure 6C,D, the current peaks vs. the square root of scan rate dependence are presented for Pt on glass and Pt on Si, respectively. A very good linear relationship (R^2^ values exceed 0.95 value) for both types of substrates indicates that the electrochemical reactions are controlled by the diffusion mechanism.

In addition, the dependence of current peaks vs. scan rate for both chips (Figure 6E,F) also shows a good linear relationship (R^2^ about 0.98) suggesting that the electrochemical reactions are also controlled by the adsorption processes.

Table 2 summarizes the analysis of the peak at −0.18 V vs. Ag/AgCl. From 30 CV measurements, the average current density and the standard deviation percentage were determined. The observed differences between the average current densities depending on the type of substrate may come from the exchange of electrolytes after the measurements. These differences in average current density are significant, the maximum value is close to 0.05 mA/cm^2^ in the case of the highest scanning speed (200 mV/s), where the highest values of current densities were recorded. An important parameter is the standard deviation of the peak maximum, which is helpful to determine the repeatability of electrochemical measurements. Depending on the scanning speed and the type of substrate, the standard deviations range from 0.23 to 2.56%. However, the average standard deviation for the analyzed types of electrodes was estimated as 0.94% for the thin-film Pt electrode on glass and 0.97% for the Pt electrode on Si. Compared to the standard deviation values obtained on classic microelectrodes (Table 1), the standard deviations, depending on the microelectrode diameter, ranged from 0.83 to 2.58%. The results obtained on thin-film microelectrodes are satisfactory, and we believe such electrodes can be successfully used in electrochemical measurements. 

### 3.3. Electrochemical Response in Acid (0.1 M HCl)

**Figure 7:**
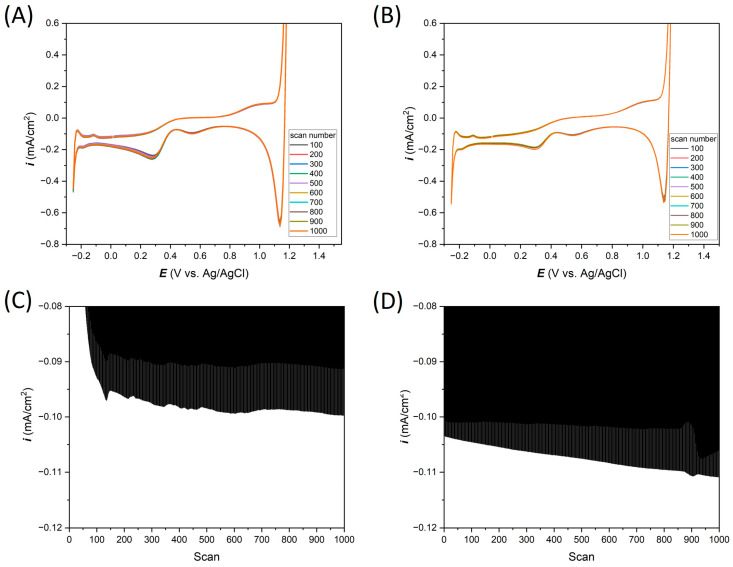
A total of 1000 cyclic voltammetry measurements in 0.1 M HCl, (**A**) Ti-Pt on glass, (**B**) Ti-Pt on oxidized Si; and cathodic peaks at 0.54 V vs. Ag/AgCl analysis for (**C**) Ti-Pt electrode on glass and (**D**) Ti-Pt electrode on oxidized Si.

Analogous electrochemical measurements, as in Section 3.2, were performed in 0.1 M HCl. In Figure 7, the long-term cyclic voltammetry results are plotted. For measurements carried out in an acid environment, CV measurements were made in the potential range from −0.25 to 1.2 V vs. Ag/AgCl (Figure 7A,B, for Ti-Pt electrode on glass and Ti-Pt electrode on Si, respectively). The collected 1000 CV cycles show very stable electrochemical behavior in 0.1 M HCl, which can also be seen in the current density values of the peak at 0.54 V vs. scan number. As was previously observed, the same shape of the curves can be observed as in the case of the wire-based Pt microelectrodes. 

**Figure 8:**
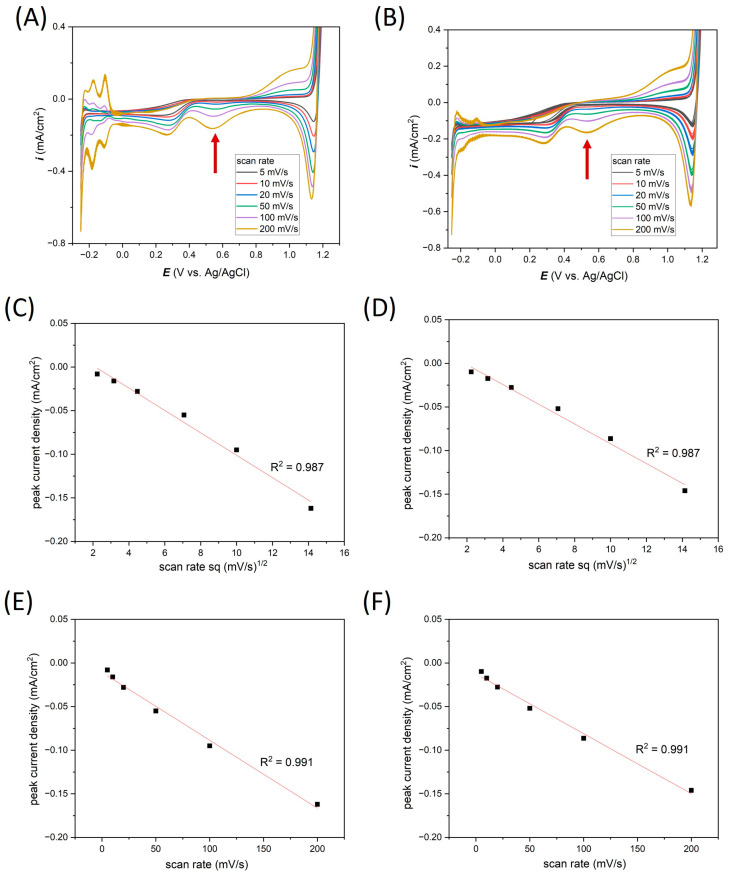
CV curves depending on scan rate rejestred in 0.1 M HCl electrolytes for (**A**) Ti-Pt on glass, (**B**) Ti-Pt on oxidized Si; peak 0.54 V vs. Ag/AgCl linearity analysis for (**C**,**E**) Ti-Pt on glass and (**D**,**F**) Ti-Pt on oxidized Si. Red arrows indicate peak, that were analysed.

By the analogy to the measurements in the previous section, in Figure 8C–F, the relationship between the cathodic current peaks read from the 20th CVs at the potential 0.54 V vs. Ag/AgCl relative to the scanning speed for Pt on glass and Pt on Si is plotted. 

In 0.1 M HCl, a very good linear relationship (R^2^ values are equal for Pt on glass and Pt on Si) indicates that the electrochemical reactions are controlled both by the diffusion and adsorption mechanisms.

Table 3 presents the analysis of the current densities recorded in the 0.54 V peak vs. Ag/AgCl in 0.1 M HCl solution for the Ti-Pt microelectrodes on two substrates depending on the scan rate. In general, in the case of measurements with a higher scan rate (from 50 to 200 mV/s), there were no significant differences in the average values of current densities depending on the type of substrate. The maximum observed difference is 0.01 mA/cm^2^. Also, in these cases, the standard deviation was less than 1%. However, in the measurements with a lower scan rate, peaks with a very low current density were registered, which was the reason for the standard deviation reaching up to 25% at a scan rate of 5 mV/s for the thin-film Ti-Pt microelectrode on glass. Mean standard deviations from the measurements for all scanning rates were 6.10 and 2.79%, respectively, for the Ti-Pt electrode on glass and Si. Considering the higher scanning rates of 50 to 200 mV/s, the average values for Ti-Pt on glass and oxidized Si were 0.75 and 0.80%, respectively.

### 3.4. Electrochemical Response in Alkaline (0.1 M KOH)

**Figure 9:**
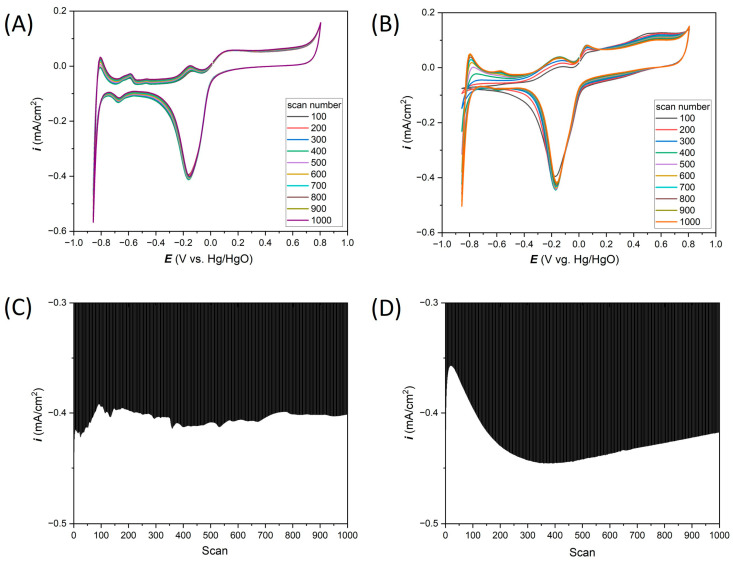
A total of 1000 cyclic voltammetry measurements in 0.1 M KOH, (**A**) Ti-Pt on glass, (**B**) Ti-Pt on oxidized Si; and cathodic peaks at −0.18 V vs. Ag/AgCl analysis for (**C**) Ti-Pt on glass and (**D**) Ti-Pt on oxidized Si.

As both glass and Si substrates can be susceptible to alkaline, analyzing the electrochemical response in KOH was relevant. Measurements for 0.1 M KOH electrolyte were carried out in the same way as for salt and acid. In Figure 9, the long-term cyclic voltammetry results are presented. For measurements carried out in an alkali environment, CV measurements were made in the potential range from −0.85 to 0.8 V vs. Hg/HgO (Figure 9A,B, for Ti-Pt on glass and Ti-Pt on Si, respectively). The obtained 1000 CVs show stable electrochemical behavior for Ti-Pt on glass, while for Ti-Pt on Si, there are some differences observed in the CV curves.

**Figure 10:**
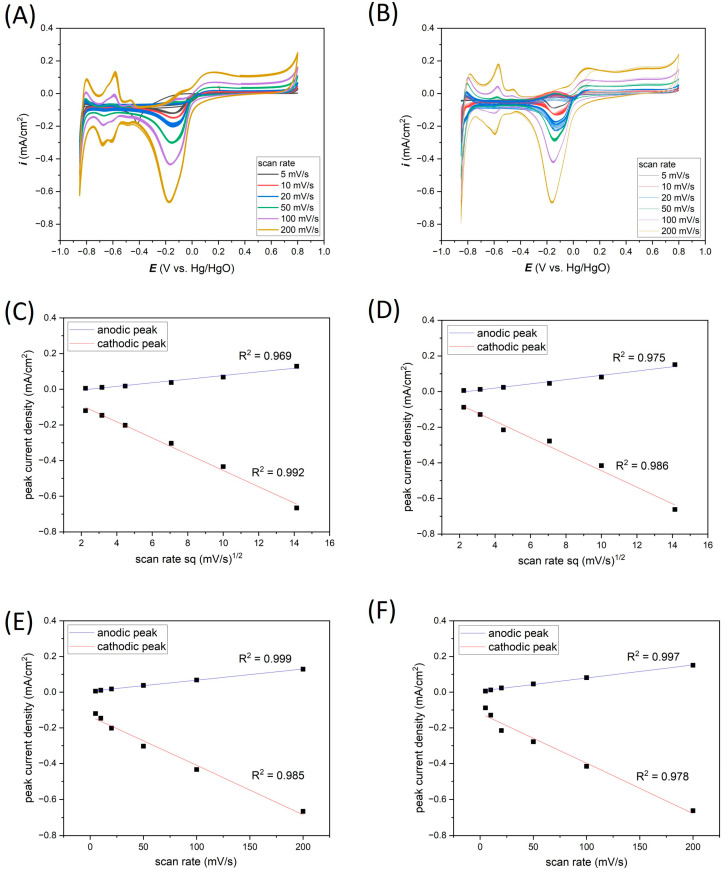
CV curves depending on scan rate registered in 0.1 M KOH electrolytes for (**A**) Ti-Pt on glass, (**B**) Ti-Pt on oxidized Si; and anodic/cathodic peaks linearity for (**C**,**E**) Ti-Pt on glass and (**D**,**F**) Ti-Pt on oxidized Si.

In Figure 10, the relationship between the cathodic current peaks read from 20th CVs at the potential −0.15 V vs. Hg/HgO and the anodic current peak at the potential close to 0.15 V vs. Hg/HgO relative to the scanning speed revealed a good linear relationship (R^2^ values are similar to those obtained in salt and acid), indicating that the electrochemical reactions are controlled both by diffusion and adsorption mechanisms.

Analogously to the measurements carried out in the neutral and acidic environments, series were made in a 0.1 M KOH solution, and the results obtained are presented in Table 4. As noted previously, larger standard deviations were noted for lower scan rate values (5, 10, and 20 mV/s). The average standard deviations for all measurements were determined to be 2.16% for Ti-Pt on glass, while for Ti-Pt on oxidized silicon substrate, they were 2.77%.

### 3.5. SEM Analysis

**Figure 11:**
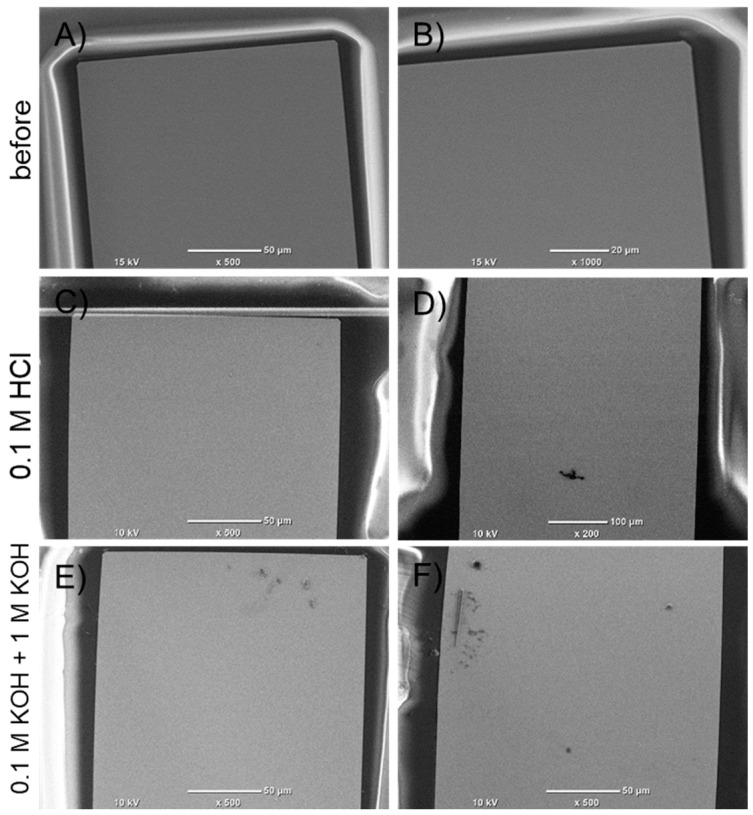
SEM pictures of Ti-Pt electrode on glass substrate: (**A**,**B**) before the tests, (**C**,**D**) after measurements in 0.1 M HCl, (**E**,**F**) after measurements in 0.1 M KOH.

**Figure 12:**
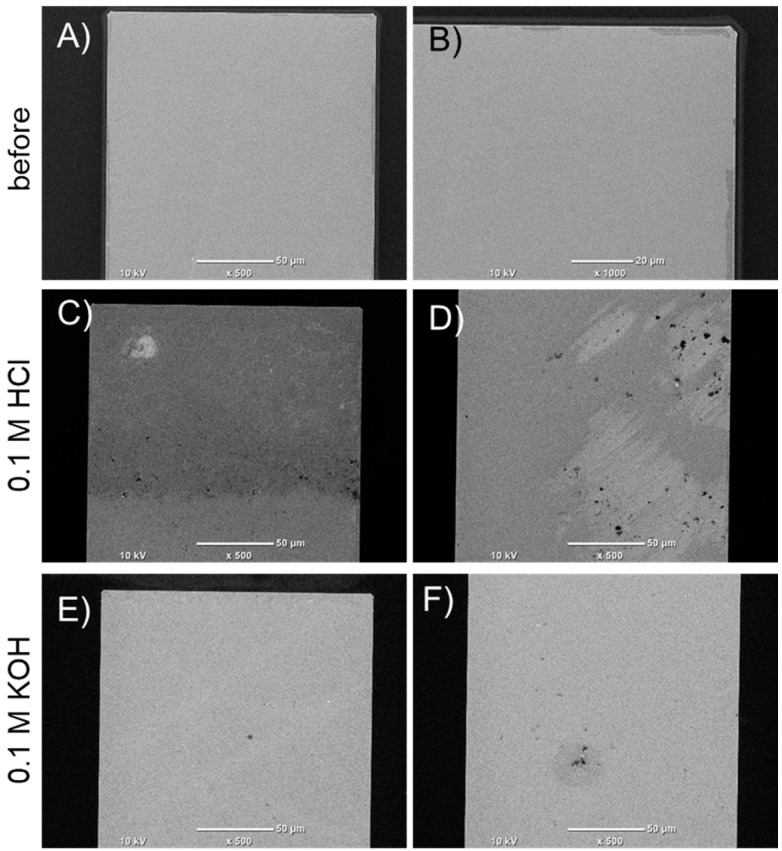
SEM pictures of Ti-Pt electrode on oxidized silicon substrate: (**A**,**B**) before the tests, (**C**,**D**) after measurements in 0.1 M HCl, (**E**,**F**) after measurements in 0.1 M KOH.

SEM was used to evaluate the surface quality of electrodes before and after electrochemical processes. The aim of the present study was to verify that the thin-film Ti-Pt microelectrodes possess sufficient stability, the films should be characterized by good adhesion, and there should be no defects in morphology. During the analysis, the focus was on the middle part and the tip of the electrode line; the quality of electrode surfaces and edges was evaluated. In Figure 11 and Figure 12, SEM pictures of thin-film Ti-Pt electrodes on glass and Si are presented, respectively.

The surface of the Ti-Pt electrode on glass is smooth. The electrode lines have sharp edges and good adhesion. After the testing, the electrodes still had a similar surface, without significant defects, as shown in Figure 11.

The analysis of the thin-film Ti-Pt electrodes on oxidized Si (Figure 12) also indicated a satisfactory smoothness and sharp edges. Only slight irregularities were recorded near one edge of the electrode, but the film was continuous (Figure 12B). After electrochemical measurements, some damage of the thin film was observed. The defects were local, and the electrode was continuous (Figure 12C,D). 

Table 5 presents a comparison of the standard deviation values for the wire-based Pt microelectrodes and the thin-film Ti-Pt microelectrodes in the solutions containing chloride ions. The values in the table represent the mean standard deviations calculated for scan rates from 5 to 200 mV/s. From 30 CV measurements at the specified scan rate, the standard deviation in the characteristic peak in the curves was calculated. Then, these values were averaged, and the standard deviation was determined for each environment and each electrode. In the case of the KCl environment, standard deviation values of over 2.5% are observed for the wire-based microelectrodes. In contrast, thin-film electrodes are characterized by a much smaller standard deviation of less than 1%. In the HCl acid solution, higher values of the standard deviation by several percent are observed. In the KOH electrolyte, the standard deviation is over 4% for wire-based microelectrodes and over 2% for thin-film electrodes. This confirms that thin-film Pt microelectrodes are stable, have good repeatability of electrochemical measurements, and generally perform better than wire-based microelectrodes.

## 4. Conclusions

In this work, the electrochemical stability and repeatability of two types of Pt electrodes—classical, wire-based, and thin-film, microfabricated—were assessed and compared. Electrochemical measurements were performed on the thin-film electrodes in the selected inorganic electrolytes (KCl, HCl, KOH), and on the wire-based electrode (three different diameters), serving as a reference for benchmarking in the neutral electrolyte. The electrochemical tests consisted of 1000 CV measurements at a 100 mV/s scan rate and a series of 30 CV measurements at scan rates in the range 5–200 mV/s. The peaks in the CV curves were defined. For selected peaks, the average current density and standard deviation were determined, which allowed us to evaluate the repeatability of measurements. The electrochemical stability was determined during measurements of 1000 CV, and the changes in the current density of selected peaks were observed. In the case of classical Pt microelectrodes, the average standard deviation of the change in current density was 0.83 to 2.58%. In the same environment (0.1 M KCl), Ti-Pt values on glass and Ti-Pt on oxidized Si were equal to 0.94% and 0.97%, respectively. For measurements in HCl, there was a visible difference in the mean standard deviation depending on the scan rate. Standard deviations reached several percent in the case of low scan rate values. For faster scans (50–200 mV/s) for Ti-Pt on glass and Ti-Pt on oxidized Si, the average standard deviations were estimated to be less than 1%. However, during measurements in KOH, average standard deviations of 2.16% and 2.77% were obtained for Ti-Pt on glass and Ti-Pt on oxidized Si, respectively. SEM analysis of Pt electrodes showed good integrity of the electrodes’ surfaces. To sum up, it was found that Ti-Pt thin-film microelectrodes deposited on glass and Si substrates are a good alternative to classical microelectrodes. Thus, the thin-film technology used, as well as the material system used, provide a good basis for work on the transfer of electrochemical cells to the chip platform.

## Figures and Tables

**Table 1:** Average density calculation of peak −0.18 V vs. Ag/AgCl analysis for different wire-based Pt microelectrodes, depending on scan rate in 0.1 M KCl.

		5 mV/s	10 mV/s	20 mV/s	50 mV/s	100 mV/s	200 mV/s
50 µm Pt	Average i (mA/cm^2^)	−2.02	−2.63	−3.18	−4.52	−6.23	−8.66
	std. Dev (%)	5.45	1.18	0.54	0.38	0.30	0.74
100 µm Pt	Average i (mA/cm^2^)	−0.97	−1.14	−1.52	−2.33	−3.38	−4.88
	std. Dev (%)	11.50	1.69	0.91	0.70	0.42	0.29
200 µm Pt	Average i (mA/cm^2^)	−0.47	−0.62	−0.84	−1.31	−1.88	−2.74
	std. Dev (%)	2.85	0.68	0.36	0.45	0.32	0.30

**Table 2:** Average density calculation of peak −0.18 V vs. Ag/AgCl analysis in 0.1 M KCl for different thin-film Ti-Pt microelectrodes, depending on scan rate.

		5 mV/s	10 mV/s	20 mV/s	50 mV/s	100 mV/s	200 mV/s
Ti-Pt on glass	Average i (mA/cm^2^)	−0.13	−0.17	−0.23	−0.36	−0.53	−0.80
	Standard deviation (%)	0.38	2.56	1.74	0.43	0.30	0.23
Ti-Pt on Si	Average i (mA/cm^2^)	−0.12	−0.15	−0.20	−0.33	−0.49	−0.75
	Standard deviation (%)	1.27	0.95	0.54	1.30	1.08	0.65

**Table 3:** Average density calculation of peak 0.54 V vs. Ag/AgCl analysis for thin-film Ti-Pt microelectrodes on glass and oxidized silicon substrates in 0.1 M HCl, depending on scan rate.

		5 mV/s	10 mV/s	20 mV/s	50 mV/s	100 mV/s	200 mV/s
Ti-Pt on glass	Average i (mA/cm^2^)	−0.02	−0.03	−0.04	−0.06	−0.09	−0.16
	Standard deviation (%)	25.24	5.52	3.75	0.86	0.76	0.60
Ti-Pt on Si	Average i (mA/cm^2^)	−0.05	−0.06	−0.06	−0.07	−0.09	−0.15
	Standard deviation (%)	12.78	1.24	0.34	0.71	0.84	0.86

**Table 4:** Average density calculation of peak −0.18 V vs. Hg/HgO analysis for different Ti-Pt electrodes in 0.1 M KOH, depending on scan rate.

		5 mV/s	10 mV/s	20 mV/s	50 mV/s	100 mV/s	200 mV/s
Ti-Pt on glass	Average i (mA/cm^2^)	−0.12	−0.15	−0.20	−0.30	−0.43	−0.66
	Standard deviation (%)	6.96	0.78	3.84	0.69	0.33	0.36
Ti-Pt on Si	Average i (mA/cm^2^)	−0.09	−0.13	−0.19	−0.28	−0.42	−0.66
	Standard deviation (%)	1.03	2.08	1.58	1.72	0.68	0.41

**Table 5:** Comparison of standard deviation values in measurements carried out on platinum electrodes in various environments.

Environment	KCl	HCl	KOH
Electrode	Standard Deviation [%]		
50 µm Pt	1.43	5.67	4.14
100 µm Pt	2.58	4.80	4.92
200 µm Pt	0.83	2.44	4.02
Ti-Pt on glass	0.94	6.10	2.16
Ti-Pt on Si	0.97	2.79	2.77

## Data Availability

Data are contained within the article.

## Supplementary Materials

The following supporting information can be downloaded at: https://www.mdpi.com/article/10.3390/ma17061352/s1.
