# Supplementary figures and images for: A Study of the Long-Term Electrochemical Stability of Thin-Film Titanium–Platinum Microelectrodes and Their Comparison to Classic, Wire-Based Platinum Microelectrodes in Selected Inorganic Electrolytes
Source: Materials (Basel). 2024 Mar 15;17(6):1352. doi: 10.3390/ma17061352 (PMC10971937; doi:10.3390/ma17061352)

## Slide 1
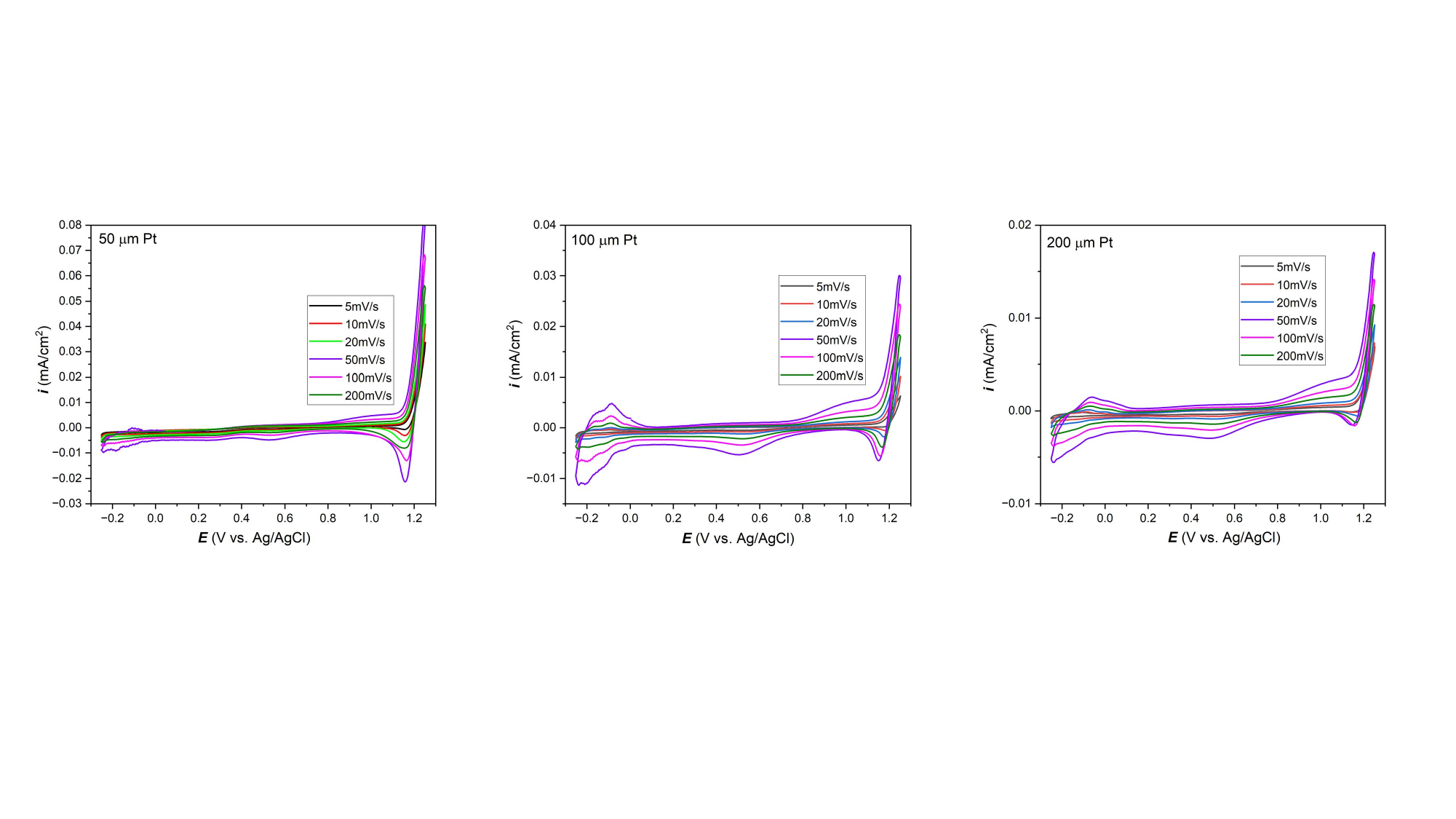

## Slide 2
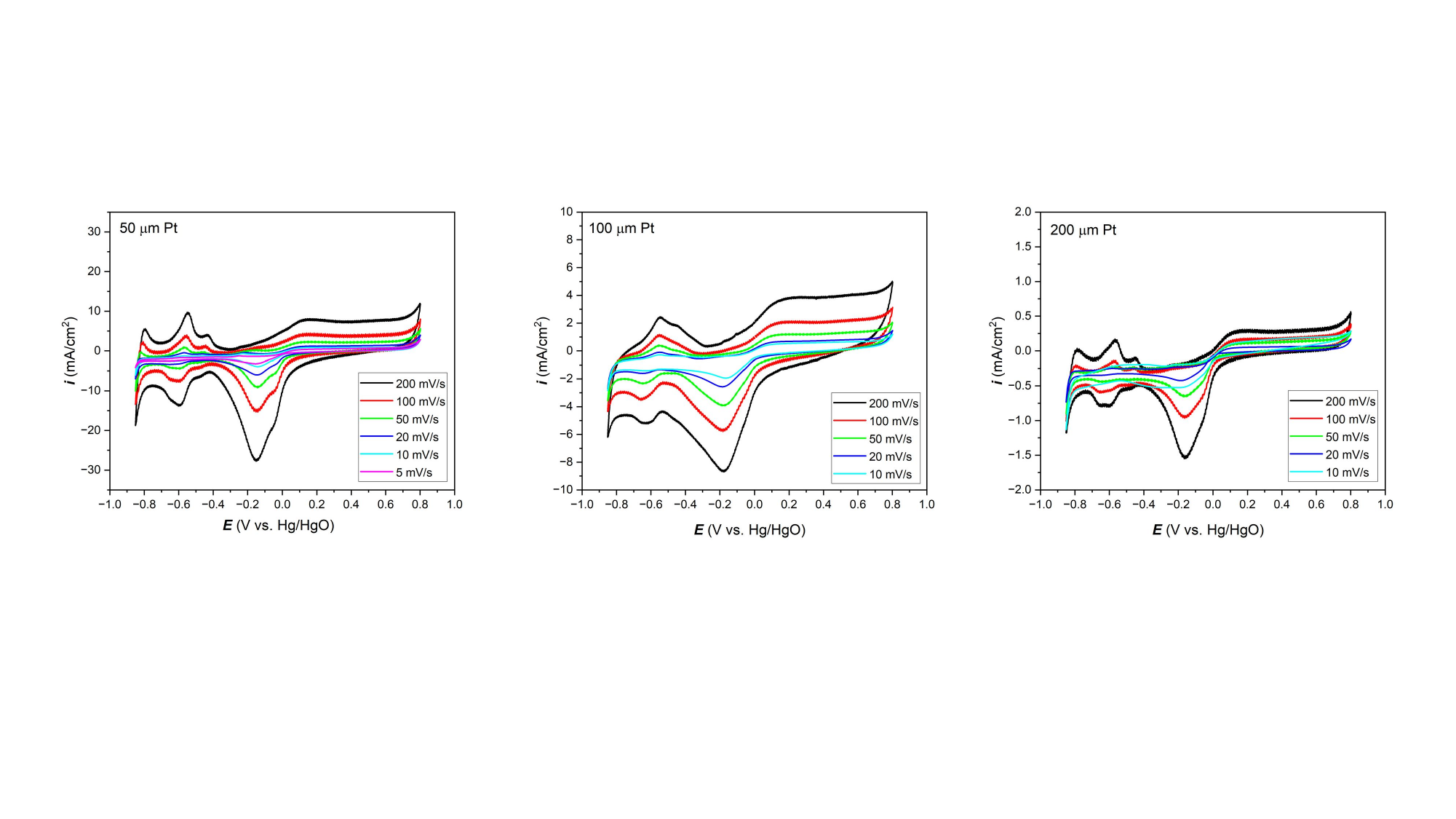

Supplement: Supplementary file 1 [file materials-17-01352-s001.zip › materials-2882736-supplementary.pptx]
